# Rapid detection of cytochrome *cd1*-containing nitrite reductase encoding gene *nirS* of denitrifying bacteria with loop-mediated isothermal amplification assay

**DOI:** 10.1038/s41598-020-73304-9

**Published:** 2020-10-05

**Authors:** Xuzhi Zhang, Qianqian Yang, Qingli Zhang, Xiaoyu Jiang, Xiaochun Wang, Yang Li, Jun Zhao, Keming Qu

**Affiliations:** 1 Yellow Sea Fisheries Research Institute, Chinese Academy of Fishery Sciences, Qingdao, 266071 China; 2 Laboratory for Marine Fisheries Science and Food Production Processes, Pilot National Laboratory for Marine Science and Technology (Qingdao), Qingdao, 266071 China; 3 College of Marine Sciences, Shanghai Ocean University, Shanghai, 201306 China

**Keywords:** Biochemical assays, Water microbiology

## Abstract

The cytochrome *cd1*-containing nitrite reductase, *nirS*, plays an important role in biological denitrification. Consequently, investigating the presence and abundance of *nirS* is a commonly used approach to understand the distribution and potential activity of denitrifying bacteria, in addition to denitrifier communities. Herein, a rapid method for detecting *nirS* gene with loop-mediated isothermal amplification (LAMP) was developed, using *Pseudomonas aeruginosa* PAO1 (*P. aeruginosa* PAO1) as model microorganism to optimize the assay. The LAMP assay relied on a set of four primers that were designed to recognize six target sequence sites, resulting in high target specificity. The limit of detection for the LAMP assay under optimized conditions was 1.87 pg/reaction of genomic DNA, which was an order of magnitude lower than that required by conventional PCR assays. Moreover, it was validated that *P. aeruginosa* PAO1 cells as well as genomic DNA could be directly used as template. Only 1 h was needed from the addition of bacterial cells to the reaction to the verification of amplification success. The *nirS* gene of *P. aeruginosa* PAO1 in spiked seawater samples could be detected with both DNA-template based LAMP assay and cell-template based LAMP assay, demonstrating the practicality of in-field use.

## Introduction

Denitrification that involves the reduction of nitrate to gaseous forms is a globally important process with relevance to many environments^1–3^. For example, denitrification can lead to the loss of nitrogen content in agricultural soils, but is also employed to remove excess nitrogen in environments like wastewaters and sludges^2^. Microorganism-mediated activities play an important role in denitrification and have even been applied to alleviate eutrophication^1,4,5^. Thus, a more detailed understanding of denitrifying organisms will aid in the application of numerous denitrification-related processes. Denitrifying bacteria comprise a wide diversity of microbial species. Cultivation-independent investigation of denitrifiers has been commonly used and has focused on analyzing key reductase functional genes^2–6^. In particular, the key step in denitrification is the reduction of nitrite to nitric oxide that is catalyzed by two structurally different, but functionally equivalent, forms of nitrite reductase encoded by the *nirK* and *nirS* genes^2,3,7^. Thus, *nir* genes are commonly used molecular markers for characterizing the diversity and abundance of denitrifying bacteria in environmental communities^3,7–9^. Of these, *nirS* is frequently used because its phylogenetic signal is largely congruent with that of 16S rRNA genes at the family or genus levels^10,11^.

The application of modern molecular biological techniques has greatly facilitated the detection of specific genes. In the last few decades, numerous methods including polymerase chain reaction (PCR)^2,3,11–15^, denaturing gradient gel electrophoresis^2,16^ and gene chips^17^ have been used to detect and analyse *nirS* gene prevalence and diversity. Among these, PCR-based methods have been prominently employed due to their high degree of accuracy and reliability. In particular, quantitative real-time PCR (qPCR) is a highly sensitive and popular tool for *nirS* detection that allows simultaneous quantification^11^. However, qPCR suffers from several drawbacks including the requirement of specialized equipment, trained operators, and high costs that largely limit its application in resource-limited settings and, especially, in-filed applications^18,19^.

Loop-mediated isothermal amplification (LAMP) that was established by Notomi et al*.*^20^ has the potential to overcome drawbacks associated with conventional PCR and revolutionize molecular biology. Compared to conventional PCR methods, it exhibits several significant advantages^18,21^ including: (1) Specialized equipment is not necessary due to the avoidance of thermal cycling, resulting in advantages including ease of miniaturization, low energy consumption, and high efficiency^20,22^. (2) Higher specificity by LAMP is achieved due to the use of four to six different primers that bind specific sites on the template strand. (3) Sensitivity is less affected by substances that usually inhibit PCR reactions^22,23^. These advantages suggest that simple assays could be developed using LAMP with elimination of the most cumbersome steps of sample pretreatment including DNA extraction and purification^24–26^. Several studies have demonstrated the capacity of LAMP to directly amplify target genes from rapidly processed, crude sample matrices^27–30^ including unprocessed samples with or without simple mechanical-based pretreatments^26,31,32^. Consequently, the employment of LAMP considerably reduces the cost and turnaround time associated with gene detection. However, there have been no reports of *nirS* gene detection via LAMP.

We have successfully used LAMP assays previously to detect *malB* genes of *Escherichia coli* (*E. coli*)^33,34^. Herein, we developed a rapid, easy-to-use, and cost-effective approach for realizing in-field detection of *nirS* gene of *P. aeruginosa*^35^, by constructing a DNA-template based LAMP assay and a cell-template based LAMP assay. The sensitivity and specificity of the new approach were characterized and compared to conventional PCR methods via a sensitivity analysis with extracted genomic DNA as template. Moreover, the LAMP assays were also used to detect *nirS* gene in seawater samples spiked with genomic DNA or *P. aeruginosa* PAO1 cells.

## Materials and methods

### Bacterial strains

Standard bacterial strains of *P. aeruginosa* PAO1 (ATCC15692), *P. aeruginosa* (ATCC9027), *P. aeruginosa* (BNCC338118), *P. aeruginosa* (BNCC125486), *P. aeruginosa* (BNCC221886), *Paracoccus denitrificans* (*P. denitrificans*, BNCC135114), *P. denitrificans* (BNCC197942), *Pseudomonas stutzeri* (*P. stutzeri*, BNCC139708), *Pseudomonas putida* (*P. putida*, BNCC337007), *Alcaligenes faecalis* (*A. faecalis*, ATCC8750), *Blastobacter denitrificans* (*B. denitrificans*, ATCC43295), *E. coli* (ATCC35150), *E. coli* (BNCC133264), *Staphylococcus aureus* (*S. aureus*, ATCC25923), *Listeria monocytogenes* (*L. monocytogenes*, ATCC19116), *Salmonella typhimurium* (*S. typhimurium*, ATCC14028), *Vibrio parahaemolyticus* (*V. parahaemolyticus*, ATCC 17802), *Vibrio cholera* (*V. cholera*, BNCC232030), and *Shigella flexneri* (*S. flexneri*, CGMCC11868) were all purchased from BIOBW Biotechnology Co., Ltd (Beijing, China). Additional strains including *E. coli* (120303502, 120303510, 120303512) and *Streptomyces* (1203EC1070400021, 1203SPL070400003, SAHL070400003) were isolated and identified from environmental samples taken from farms. *Halomonas alkaliphila* strains (strains X1, X2, X3) were isolated and identified from seawater samples. Note, unless otherwise indicated, *P. aeruginosa* in this paper referred to PAO1 (ATCC15692) strain.

### Cultivation and cell quantification

Luria–Bertani (LB) medium was used to culture *P. aeruginosa*, *E. coli*, *S. aureus*, *S. typhimurium*, *Streptomyces* spp. and *S. flexneri*. Other denitrifying strains used in this study were cultured aerobically in nutrient medium^2^. Listeria Enrichment medium was used to culture *L. monocytogenes*. Alkaline peptone water was used to culture *V. cholera*. While 2216E medium (a common complex culture medium for marine bacteria, consisting of 0.5% tryptone, 0.1% yeast extract, 3.4% NaCl and 0.01% FePO_4_, pH 7.6–7.8) was used to culture *V. parahaemolyticus* and *H. alkaliphila*. Culture media were all purchased from the Hope Bio-Technology Co., Ltd (Qingdao, China). Bacterial cultivation was conducted in accordance with previously described methods^36,37^ with minor modifications. Briefly, strains were stored at − 80 °C and then pre-grown overnight in the appropriate medium with constant shaking. The incubation temperature was 37 °C unless otherwise indicated. Active strains were then further transferred to new culture medium. After a second incubation for ~ 10 h, cell numbers were measured using a plate counting method that we have used previously^37^. The cultures were then immediately diluted to achieve the desired cell concentrations for further use, or otherwise centrifuged to collect cells for DNA extraction.

### Genomic DNA extraction and purification

DNA was extracted from cells collected from 50 mL of sub-cultured medium, followed by DNA purification using previously described methods^34^. Briefly, cells were pre-separated by centrifugation and genomic DNA was extracted and purified from the collected cells using a rapid commercial genomic DNA extraction kit (Biomed Co., Beijing, China) according to the manufacturer’s instructions. Purified DNA was then quantified using a Biodropsis BD-2000 spectrophotometer (Biodropsis Technologies Co., Ltd, Beijing, China). Template genomic DNAs were then stored in Tris–EDTA buffer (pH 7.0) at − 20 °C until further use no later than 4 weeks after extraction.

### LAMP assays

#### Primer design and synthesis

**Figure 1:**
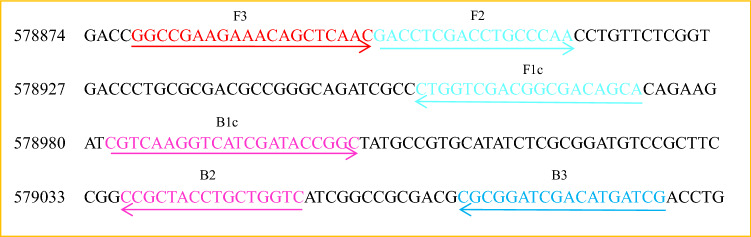
Priming locations and orientation of the LAMP primers developed to amplify *P. aeruginosa nirS*. The arrows show sequence directions from 5′ to 3′. The asterisks denote consistent nucleotides sequence not shown.

The *nirS* gene sequence of *P. aeruginosa* was obtained from the NCBI database (https://www.ncbi.nlm.nih.gov/gene/882217). LAMP primer sets to amplify *nirS* were designed based on the published DNA sequence using the LAMP primer designing software package (v.4.0, https://primerexplorer.jp/e/). Using previously published guidelines^38^, the specificity of the primers was determined using the NCBI BLAST (Basic Local Alignment Search Tool), and then screened using Primer Premier v.5.0 (PREMIER Biosoft International, Palo Alto, CA) based on the likelihood of primer dimer formation and non-specific priming. From these analyses, a single primer set was selected for LAMP assays (Fig. 1). From the first bate of F3 to the last bate of B3, there were 207 bp. The sequence of the 207 bases was checked using the NCBI BLAST. Only *nirS* gene of a dozen *P. aeruginosa* strains matched at 100%. The primers were then synthesized by Sangon Biotech Co., Ltd, (Shanghai, China). The priming locations on the target DNA sequence are shown in Fig. 1, and the primer nucleotide sequences are provided in Table 1.

**Table 1:** LAMP primer sequences.

Primer	Sequence (5′-3′)
*nirS*-F3	GGCCGAAGAAACAGCTCAAC
*nirS*-B3	CGATCATGTCGATCCGCG
*nirS*-FIP	TGCTGTCGCCGTCGACCAGTTTTGACCTCGACCTGCCCAA
*nirS*-BIP	CGTCAAGGTCATCGATACCGGCTTTTTCACCAGCAGGTAGCGG

#### LAMP reaction systems and amplification product characterization

**Figure 2:**
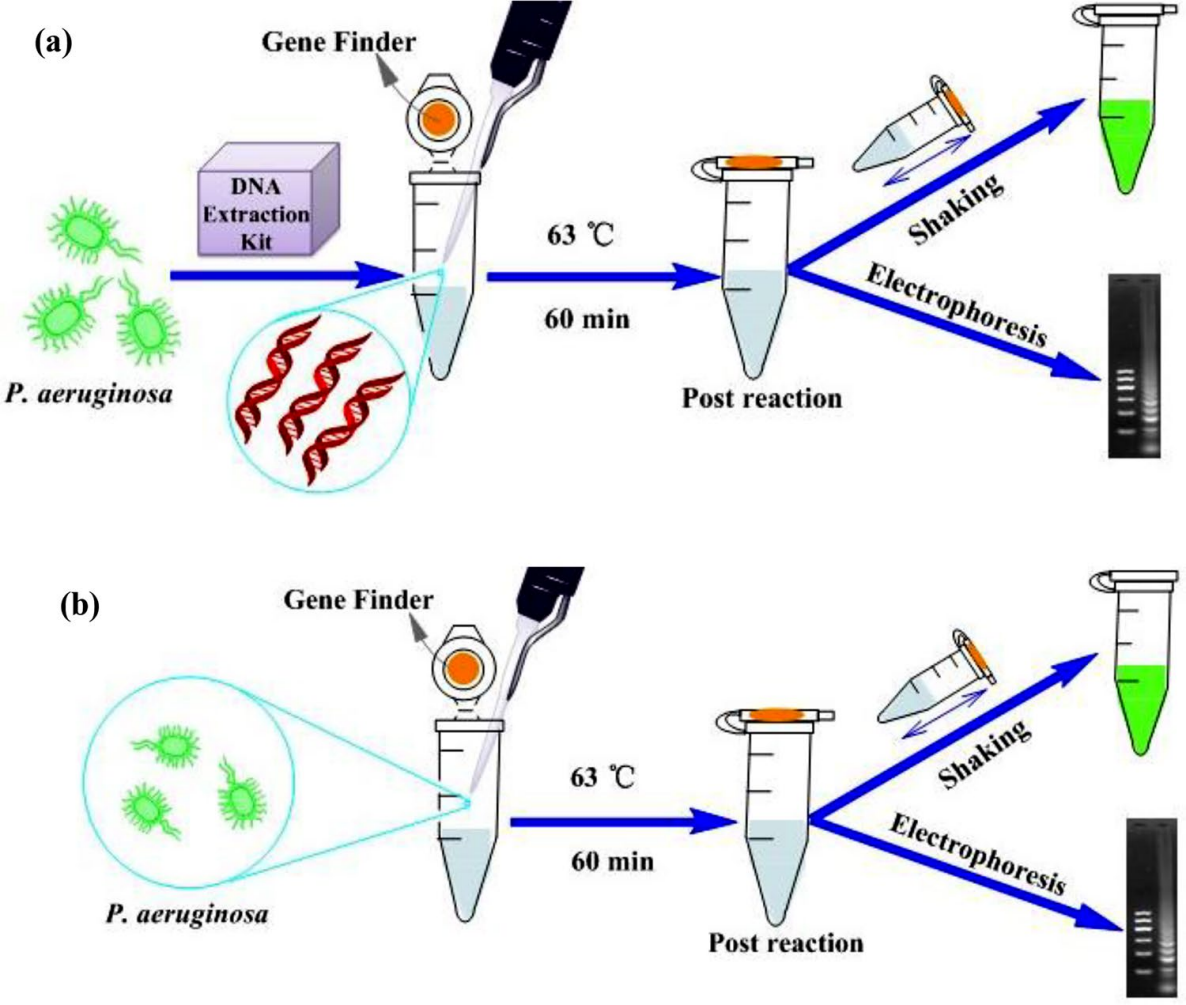
Methodological schematics for the DNA-template based LAMP assay (**a**) and the cell-template based direct LAMP assay (**b**) for detecting the *nirS* genes of *P. aeruginosa*.

As shown in Fig. 2a, LAMP assays using DNA as template, termed DNA-template based LAMP assays, were conducted using previously described methods^20,34^ with minor modifications. Unless otherwise indicated, 25 μL LAMP reaction volumes were used comprising 0.2 μM of each outer primer (B3 and F3), 1.6 μM of each inner primer (FIP and BIP), 1.2 mM of each dNTP, 2.5 μL 10 × ThermoPol® reaction buffer, 1 μL *Bst* 2.0 DNA polymerase, 6 mM MgSO_4_, and 1 μL genomic DNA template. dNTPs were purchased from MBI Fermentas (Waltham, USA) and *Bst* 2.0 DNA polymerase was purchased from New England Biolabs (Ipswich, Massachusetts, USA). Reactions were incubated at 63 °C for 60 min in a block heater, unless otherwise indicated. Based on the methods described in Tomita et al*.*^39^ LAMP reaction products were characterized by gel electrophoresis on a 2% agarose gel using a DY-6 electrophoresis apparatus (Xinghua Assay Apparatus Factory, Beijing, China) and a DNR Bio-Imaging System (MF-ChemiBis 3.2, Israel). Electrophoresis was conducted using 50 × diluted LAMP reaction products. Additional visual characterization using the fluorescent dye GeneFinder (Biov Co., Ltd., Xiamen, China) was also performed. Triplicate determinations were performed for every set of analyses.

As shown in Fig. 2b, cell-template based LAMP assays were carried out using the same method described for the *nirS* gene, but with *P. aeruginosa* cells as template rather than extracted genomic DNA. *P. aeruginosa* cells were obtained using the methods described by Kanitkar et al*.*^31^ Briefly, after the concentration of bacterial cells was quantified using the plate counting method described above, 10 mL of culture medium was centrifuged at 13,000*g* for 15 min to obtain a biomass pellet. The biomass pellet was then suspended in an appropriate volume of water and 2 μL of the bacterial suspension was immediately used as amplification template. LAMP products were again characterized by gel electrophoresis and fluorescent dye visualization as described above.

#### Optimization

The temperatures and incubation times for the LAMP assay were optimized based on the approach of Balbin et al*.*^40^ Briefly, amounts of *P. aeruginosa* genomic DNA varying from 18.70 fg to 187.00 ng were used as amplification template. LAMP was then carried out at 61 °C, 62 °C, 63 °C, 64 °C, and 65 °C. After determining the optimal temperature for the assays, LAMP was then conducted with varying incubation times including 10, 20, 30, 40, 50, 60, 70, and 80 min.

### Specificity

The specificity of the designed *nirS* primer set was determined using either genomic DNA or bacterial cells as amplification templates. For the former, ~ 0.1 ng genomic DNA from *P. aeruginosa*, *E. coli*, *S. aureus*, etc*.* was used as template for the LAMP assay. Gel electrophoresis and/or visual detection were used to characterize the amplification products. For assays with cells, ~ 10^5^ CFU of bacterial cells were used as amplification template. Unless otherwise indicated, for both sets of assays, 0.19 ng of *P. aeruginosa* genomic DNA and pure water were used as the positive and negative controls, respectively.

### Sensitivity

#### Sensitivity of DNA-template based LAMP assay

The sensitivity of the DNA-template based LAMP assay for *nirS* was determined using a tenfold serial dilution of the template DNA. Results were again characterized using both gel electrophoresis and visual detection. The limits of detection (LOD) were obtained from these analyses using the same reaction parameters discussed above. Unless otherwise indicated, each assay was conducted in triplicate.

In addition, synthetized double-stranded DNA (sequence was in Fig. 1) at known concentration (copy/μL) was also used as template for LAMP assay as we reported previously^41^, to calculate the sensitivity on copy number of *nirS* gene. The LAMP reactions were incubated at 63 °C for 60 min.

#### Sensitivity of cell-template based LAMP assay

The sensitivity of the cell-template based LAMP assay for *nirS* was determined using methods described by Lee et al*.*^27^ with minor modifications. Briefly, a biomass pellet of bacterial cells was obtained from centrifugation of the cell cultures. The pellet was then suspended in 5 mL water. An aliquot (500 μL) of the bacterial suspension was used to measure cellular concentrations. The remainder of the suspension was used as template for direct amplification using the LAMP assay with tenfold serial dilutions to identify the LOD (CFU/reaction). The results were characterized with both gel electrophoresis and visual detection. Unless otherwise indicated, each assay was conducted in triplicate.

### Conventional PCR assays

The F3 and B3 primers were used for conventional PCR assays following the methods of Verma et al*.*^19^ and Stedtfeld et al*.*^32^ PCR reactions comprised 25 μL volumes consisting of 1 μL genomic DNA template, 12.5 μL Version 2.0 Taq polymerase plus dye (TaKaRa Biotechnology Co., Ltd., Dalian, China), and 1 μL of each primer (0.2 μM in reaction mix). PCR reaction conditions consisted of 94 °C for 3 min, followed by 30 cycles of 94 °C for 45 s, 54 °C for 55 s, 72 °C for 90 s and a final extension at 72 °C for 10 min. A 5 μL aliquot of each PCR product was subjected to 2% agarose gel electrophoresis for characterization.

### Detection of nirS gene in spiked seawater samples

To investigate the ability of the LAMP assay to detect *nirS* in complex natural matrices, a spiked LAMP assay was conducted with seawater samples. The seawater sample was collected from the Yellow Sea (36° 06.54′ N; 120° 39.28′ E). Water salinity (31.01‰) and pH (8.062) were measured using a YSI 556 Multi Probe System (Envisupply Co., USA). Bacterial biomass and extracellular DNA were removed from the water using filtration with 0.22 μm Sterivex filters followed by filtration with Silicone membranes (EMD Millipore Corp., Billerica, MA), respectively^32^. The capacity of the LAMP assays to detect *nirS* gene was then challenged using seawater samples spiked with genomic DNA and *P. aeruginosa* cells, respectively. All seawater samples were used for the next step within 20 min after the spiked performance, unless otherwise indicated.

#### DNA-template based LAMP assay

Extracted *P. aeruginosa* genomic DNA was added to the filtered seawater over a concentration range of 1.27 × 10^2^–1.27 × 10^8^ fg/μL. Then, 1 μL of seawater samples with varying spiked DNA concentrations were directly used as templates for LAMP assays. The LOD were then determined based on visual detection of the amplification products.

#### Cell-template based LAMP assay

A ~ 10^13^ CFU/mL bacterial suspension was prepared in water, as described above. Bacterial suspensions were added to the filtered seawater at various volumes to generate spiked samples over a cell concentration range of 1.68 × 10^1^–1.68 × 10^7^ CFU/mL. For each spiked sample, a 50 mL cell suspension was subjected to centrifugation to pellet cells. The obtained biomass pellet was then directly used as template for LAMP assays. In addition, 2 μL of spiked seawater samples were directly used as templates for LAMP assays. The LOD were determined based on visual detection of amplification products.

## Results

### LAMP amplification of nirS

**Figure 3:**
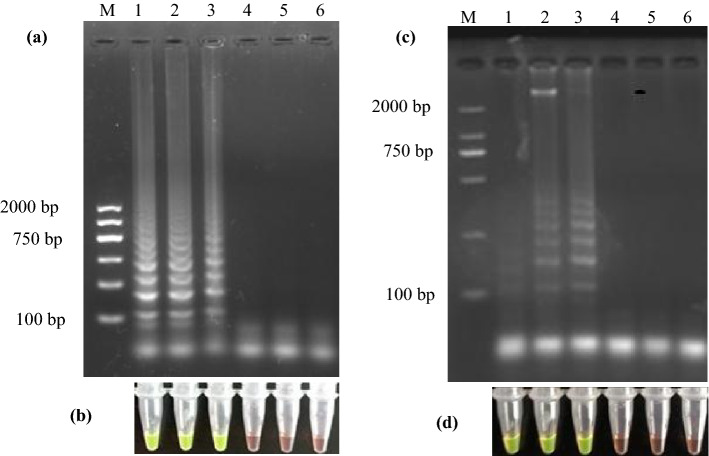
Left: Detection of the *nirS* gene with DNA-template based LAMP assay, characterizing with gel electrophoresis (**a**) and GeneFinder (**b**). Lanes 1–3 are amplification reactions using 0.19 ng of extracted genomic DNA as template; Lanes 4–6 are the negative control. Right: Detection of the *nirS* gene with cell-template based LAMP assay, characterizing with gel electrophoresis (**c**) and GeneFinder (**d**). Lanes 1–3 are amplification reactions using 3.36 × 10^2^ CFU *P. aeruginosa* cells as template; Lanes 4–6 are the negative control. The LAMP reactions were incubated at 63 °C for 60 min. In all negative control reactions, water was used as template.

Using 0.19 ng genomic DNA of *P. aeruginosa* as template, LAMP amplification of *nirS* at 63 °C for 60 min resulted in amplification products of various size, as indicated by gel electrophoresis and the presence of many sized bands in a reproducible ladder-like pattern (Fig. 3a), which was the same phenomena obtained somewhere^20,23,24,33,34^. The absence of these ladder-like patterns in the negative control indicated that the pattern was due to *nirS* amplification. Light green fluorescence of positive amplification products when using the GeneFinder dye was evident (Fig. 3b) as previously observed^33^, while negative controls remained orange. To avoid inhibition of the dye fluorescence, 1 μL of GeneFinder solution was coated inside of the Eppendorf tube cover, rather than premixing it into the LAMP reaction mixture.

To optimize the assay, LAMP reactions were conducted at various temperatures and incubation times. The ladder-like electrophoresis patterns observed in the initial amplifications were reproduced when using 0.19 pg of genomic DNA as template and incubating reactions at 63 °C for 60 min. Modifying the incubation temperatures or using incubation times < 60 min resulted in the absence of ladder-like electrophoresis band patterns. Consequently, an incubation temperature of 63 °C and time of 60 min were selected for further analyses.

Using 3.36 × 10^2^ CFU of *P. aeruginosa* cells as template, cell-template based LAMP assays were also incubated at 63 °C for 60 min and yielded similar successful amplification results as with amplification using genomic DNA (Fig. 3c,d), without negative control amplification. These results indicated positive LAMP amplification from *P. aeruginosa* cells under the specified conditions.

### Specificity of LAMP assay

The specificity of the LAMP assay for the detection of *nirS* gene was determined using ~ 0.10 ng of genomic DNA from various bacterial species as template (Table 2). Results of visual detection (Figure S1) indicated the specific amplification of *nirS* from bacterial genomic DNA that contains the cytochrome *cd1*-containing nitrite reductase encoding gene^2,35^. Moreover, no false positive or false negative results were observed when using template DNA from a wide range of control bacterial species (Figure S2), also indicating good specificity of the LAMP assay for the *nirS* gene.

**Table 2:** DNA-template based LAMP assays for detecting *nirS* gene of various bacterial species.

Species	Strain	LAMP amplification	
		Gel electrophoresis	Fluorescent dye
*P. aeruginosa*	PAO1 ATCC 15692	+	+
	ATCC 9027	+	+
	BNCC125486	+	+
	BNCC338118	+	+
	BNCC 221886	+	+
*P. denitrificans*	BNCC135114	+	+
	BNCC197942	+	+
*P. stutzeri*	BNCC139708	+	+
*P. putida*	BNCC337007	−	−
*A. faecalis*	ATCC8750	−	−
*B. denitrificans*	ATCC43295	−	−
*E. coli*	ATCC 35150	−	−
	BNCC133264	−	−
*S. aureus*	ATCC25923	−	−
*L. monocytogenes*	ATCC 19116	−	−
*S. typhimurium*	ATCC 14028	−	−
*S. flexneri*	CGMCC11868	−	−
*V. parahaemolyticus*	ATCC 17802	−	−
*V. cholerae*	BNCC232030	−	−
*H. alkaliphila*	X1	−	−
*H. alkaliphila*	X2	−	−
*H. alkaliphila*	X3	−	−
*E. coli*	120303502	−	−
*E. coli*	120303510	−	−
*E. coli*	120303512	−	−
*Streptomyces*	1203EC1070400021	−	−
*Streptomyces*	1203SPL070400003	−	−
*Streptomyces*	SAHL070400003	−	−

+ positive, − negative.

Experiments were also conducted to evaluate the specificity of *nirS* detection via cell-template based LAMP assay using ~ 10^3^ CFU/reaction from various bacterial species as template. The results from these assays (Table S1) were consistent with those obtained from DNA-template based LAMP assays, indicating high specificity of cell-template based amplification under the selected conditions.

### Sensitivity of LAMP assay

**Figure 4:**
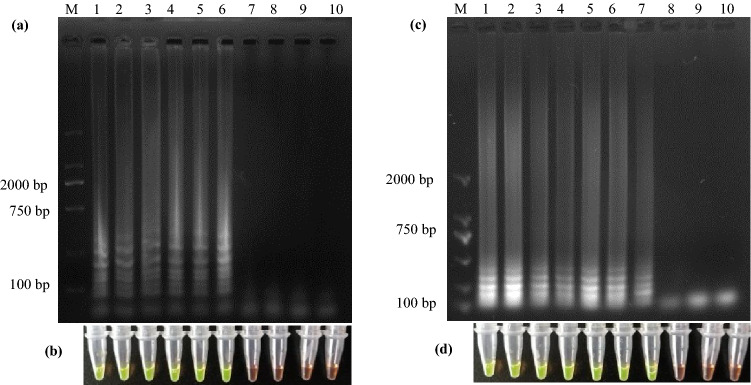
Left: DNA-template based LAMP assay results of *nirS* gene using 187.00 ng, 18.70 ng, 1.87 ng, 187.00 pg, 18.70 pg, 1.87 pg, 187.00 fg, 18.70 fg, 1.87 fg, and 0.00 fg genomic DNA as template in each reaction (from lane 1 to lane 10), characterizing with gel electrophoresis (**a**) and GeneFinder (**b**). Right: cell-template based LAMP assay results of *nirS* gene using 3.36 × 10^8^, 3.36 × 10^7^, 3.36 × 10^6^, 3.36 × 10^5^, 3.36 × 10^4^, 3.36 × 10^3^, 3.36 × 10^2^, 3.36 × 10^1^, 3.36 × 10^0^, and 0.00 CFU *P. aeruginosa* cells as template in each reaction (from lane 1 to lane 10), characterizing with gel electrophoresis (**c**) and GeneFinder (**d**).

We assessed the sensitivity of DNA-template based LAMP assay over the amount range of 1.87 fg–187.00 ng. The results (Fig. 4a,b) indicated that the LOD was 1.87 pg/reaction with these specified parameters. Below the LOD, no visual detection of amplification products was observed. Moreover, the sensitivity of gel electrophoresis and visual detection were equivalent, suggesting that they were both equally appropriate for determining LAMP amplification success. Using longer incubation times can lower the LOD of LAMP assays at the expense of analysis efficiency^42^. Consequently, 60 min was selected as the incubation time for all other reactions. In addition, when the synthetized double-stranded DNA was used as template for the assay of *nirS* gene, we obtained a LOD of 16.4 copy/μL. The sensitivity of cell-template based LAMP assays was also evaluated as above with amount of *P. aeruginosa* cells over the range of 3.36 × 10^0^–3.36 × 10^8^ CFU/reaction (Fig. 4c,d). The LOD was 3.36 × 10^2^ CFU/reaction.

### Comparison of PCR and LAMP

**Figure 5:**
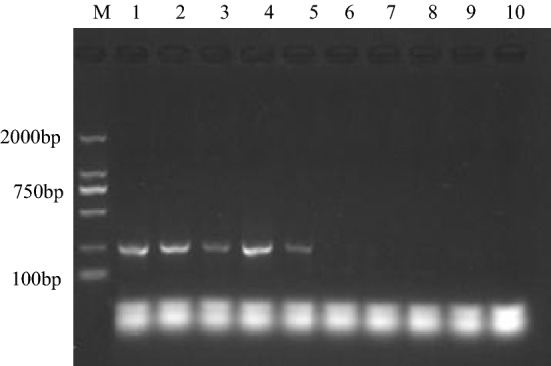
Conventional PCR assay results of *nirS* using 187.00 ng, 18.70 ng, 1.87 ng, 187.00 pg, 18.70 pg, 1.87 pg, 187.00 fg, 18.70 fg, 1.87 fg, and 0.00 fg genomic DNA of *P. aeruginosa* as template in each reaction (from lane 1 to lane 10), characterizing with gel electrophoresis.

Using the F3 and B3 primers, experiments were conducted to determine the sensitivity of conventional PCR assay in comparison with the DNA-template based LAMP assay. Genomic DNA amount ranging from 1.87 fg to 187.00 ng/reaction were used as template for the reactions. Gel electrophoresis characterization of PCR amplification products indicated no amplification when the DNA template was in a lower amount than 18.70 pg/reaction (Fig. 5), but amplification was detected over the range of 18.70 pg to 187.00 ng/reaction. These results indicate a wider dynamic range of the LAMP assays, with tenfold greater sensitivity than conventional PCR when using genomic DNA. Further, no PCR amplification was detected when *P. aeruginosa* cells were directly added to each PCR reaction mixture over the range of 3.36 × 10^8^–3.36 × 10^4^ CFU/reaction.

### Detection of nirS gene in spiked seawater samples

To investigate the ability of the DNA-template based LAMP and the cell-template based LAMP assays for detecting *nirS* in complex matrices, we spiked seawater samples with *P. aeruginosa* genomic DNA or cells over concentration ranges of 1.27 × 10^2^–1.27 × 10^8^ fg/μL and 1.68 × 10^1^–1.68 × 10^7^ CFU/mL, respectively. A 2 μL aliquot of the spiked samples was then used as a template in reactions with incubations at 63 °C for 60 min. Amplification success was characterized by staining with GeneFinder. Amplifications did not occur with genomic DNA concentrations lower than 1.27 × 10^4^ fg/μL in the spiked samples (Figure S3). In the cell-template based LAMP assay, a 50 mL mixture of seawater spiked with cells at different concentrations was pretreated by centrifugation to pellet cells. The obtained biomass pellet was then directly used as the template for the cell-template based LAMP assay. Amplifications occurred using every biomass pellet obtained from the spiked samples (Figure S4). When using a 2 μL spiked sample as a template, amplifications only occurred when cell concentrations were greater than 1.68 × 10^4^ CFU/mL (Figure S5).

## Discussion

Denitrification and denitrifying microbial communities have recently received widespread research attention due to their important contributions to the global nitrogen cycle^1,8,43^. Functional genes involved in nitrite reduction, especially the cytochrome *cd1*-containing nitrite reductase encoding gene, *nirS*, are commonly used as molecular markers to detect denitrifying populations and potential activities^8,43–46^. Concomitantly, the recent development of a novel gene amplification procedure, LAMP, has shown great promise in overcoming the numerous drawbacks of conventional PCR gene amplification methods. In this study, a DNA-template based LAMP assay and a cell-template based LAMP assay were developed to detect *nirS* gene of *P. aeruginosa*. The characteristics of these assays are discussed below and compared against those of conventional PCR assays.

LAMP reactions achieve DNA amplifications using a one-step reaction with a set of target-specific primers (e.g., FIP, BIP, F3, and B3) that recognize six distinct sites flanking the target sequence. The FIP and BIP, each of which contains two functional sequences (one for priming extension in the first stage and the other for self-priming in the second stage) corresponding to the sequences (sense and antisense) of the target dsDNA, play major roles in the LAMP reaction. Catalyzing by *Bst* DNA polymerase with strand displacement activity, LAMP reaction includes two stages. In the first stage, all of four primers are used to start structure-produce. In the second stage, only FIP and BIP are required for realizing cycling amplification. In brief, an ssDNA is released by strand displacement DNA synthesis primed by an F3 and then acts as the template for DNA synthesis primed by both BIP and B3, producing a stem-loop DNA structure. After initiation by one inner primer complementary to the loop on the product, the cycling amplification process is continued by each inner primer alternately. Thus, the specificity is higher than PCR and the final products are stem-loop DNAs with different inverted target repeats and cauliflower-like structures with multiple loops, which are ladder-like patterns characterized by gel electrophoresis^18,20^. *NirS* gene is absent in *S. aureus* and *E. coli* genomes, but present in those of *P. aeruginosa*^47^, which is consistent with other reports^35,42^. Our amplification results from LAMP specificity assays are consistent with these reports.

PCR activity strongly depends on the cycling of working temperatures, consequently requiring sophisticated equipment to accurately control reaction temperatures. One of the major advantages of the LAMP assay over conventional PCR is eliminating the need for cycling of temperatures, thereby allowing the use of simple, miniature, and affordable amplification devices, in addition to requiring lower energy consumption^22^. These features render LAMP assays suitable for use in resource-limited rural areas. Moreover, these advantages make LAMP a promising approach for realizing in-field and rapid detection and avoiding cumbersome transportation from sampling sites to specialized laboratories, as is necessary for conventional PCR detection of *nirS* gene from environmental samples^3–5,11–14^.

PCR products are typically characterized by gel electrophoresis^5,13,45^or otherwise via quantification with fluorescent probes^5,44,45^. In contrast, more quantification approaches can be employed to determine LAMP product amplification, including both endpoint and online patterns. Gel electrophoresis and GeneFinder characterization are both endpoint analyses that are appropriate for LAMP detection, as shown here and elsewhere. In addition, several alternative endpoint methods can be used, including assays with SYBR Green I, Quant-iT PicoGreen, and polyethylenimine, among others. Further, the large amount of white precipitate that is the product of insoluble magnesium pyrophosphate can be used to determine LAMP reaction success with or without centrifugation^22^. Online characterization methods can also be used to assess LAMP amplification success including the use of turbidimeters, optical fibers, or spectrophotometers that can monitor LAMP reaction progress based on the formation of magnesium pyrophosphate^21,22,34^. Consequently, the addition of special indicator reagents is unnecessary, further reducing reagent and labor costs. Importantly, instruments for real-time monitoring of LAMP amplification are already commercially available.

The results reported here indicate that conventional PCR assays of *nirS* gene required more than 18.7 fg of template DNA for each reaction, which is consistent with results from Li et al.^47^ In contrast, the LAMP assay results reported here demonstrate a LOD of 1.87 pg/reaction, indicating a significantly higher sensitivity than conventional PCR, which agrees with previous reports^19,28^. Moreover, *nirS* gene detection with conventional PCR assays required cell lysis and subsequent DNA extraction^5,45^. Consistent with these observations, we found that PCR amplification could not occur using bacterial cells as the amplification template. DNA extraction, PCR reactions, and electrophoresis typically require > 1 h each, and all of these procedures require bulky, specialized equipment. Performing real-time quantitative PCR is much quicker than traditional PCR due to the measurement of reaction results in real time. However, qPCR necessitates expensive probes, even more sophisticated equipment than traditional PCR and is still time consuming. Consequently, conventional and real-time PCR assays are not amenable to detection of *nirS* gene in point-of-care settings. LAMP has the potential to circumvent these problems due to a reduced dependence on pretreatment of samples and the ability to conduct LAMP under isothermal condition^18,22^. In particular, the efficacy of cell-template based LAMP assay considerably enhances its application in point-of-care settings^26,31,32^. For example, we successfully detected *nirS* gene of *P. aeruginosa* cells over a range of 3.36 × 10^2^–3.36 × 10^8^ CFU/reaction. These results further confirm that LAMP assays are less affected by substances that typically inhibit conventional PCR^23,34^. Consequently, simpler LAMP assays can be developed by eliminating the DNA extraction step that is necessary prior to conventional PCRs. Further, only 1 h was needed from the addition of template bacterial cells to amplification verification without the need for bulky and sophisticated equipment. Moreover, *nirS* gene of *P. aeruginosa* could be detected in spiked seawater samples with either DNA template or bacterial cells template, further demonstrating the practicality of the LAMP assays, even in complex background matrices. It should be noted, however, that sensitivity of the LAMP assay was clearly affected by the presence of complex co-existing substances in the seawater.

Future investigations of *nirS* amplification via LAMP assays will focus on improving the assays through three target areas. First, the specificity of the LAMP assay towards *nirS* from more taxa will be tested to determine its capacity for analyzing denitrifier communities, in general. Second, methods will be developed to eliminate interference from dead cells and extracellular DNA, because only gene expression from viable cells is meaningful towards understanding functional protein expression and consequent denitrification activity. Lastly, a quantitative LAMP assay will be developed to determine the relationship between *nirS* gene copy abundance in viable microbial cells and denitrifying efficiency.

## Conclusions

Herein, a rapid and specific detection of *nirS* gene with LAMP assay was developed for the first time, using the gel electrophoresis or GeneFinder visualization to characterize amplification products. Under optimized conditions, the LOD of DNA-template based LAMP assay was 1.87 pg/reaction, which was an order of magnitude lower than that obtained by conventional PCR assays; while the LOD of cell-template based LAMP assay was 3.36 × 10^2^ CFU/reaction. Only 1 h was needed from the addition of bacterial cells to the reaction to the verification of amplification success, requiring no bulky and sophisticated equipment. Their practicality using environmental samples was preliminarily demonstrated using seawater samples spiked with genomic DNA or *P. aeruginosa* cells. Overall, the LAMP assays presented here, particularly the cell-template based one, were superior to conventional PCR assays in terms of sensitivity, specificity, turnover-time, simplicity, and cost. Importantly, they are ready for in-field applications.

## Supplementary information

Supplementary Information.
